# Clinical Course and Impact of Immune Checkpoint Inhibitor Colitis Resembling Microscopic Colitis

**DOI:** 10.1093/crocol/otac008

**Published:** 2022-03-15

**Authors:** Thomas W Fredrick, Guilherme P Ramos, Manuel B Braga Neto, Sunanda Kane, William A Faubion, Edward V Loftus, Darrell S Pardi, Shabana F Pasha, Francis A Farraye, Lizhi Zhang, Laura E Raffals

**Affiliations:** 1 Department of Internal Medicine, Mayo Clinic, Rochester, Minnesota, USA; 2 Division of Gastroenterology and Hepatology, Mayo Clinic, Rochester, Minnesota, USA; 3 Division of Gastroenterology and Hepatology, Mayo Clinic, Phoenix, Arizona, USA; 4 Division of Gastroenterology and Hepatology, Mayo Clinic, Jacksonville, Florida, USA; 5 Division of Anatomic Pathology, Mayo Clinic, Rochester, Minnesota, USA

**Keywords:** colitis, microscopic, immune checkpoint inhibitors, drug-related side effects and adverse reactions, inflammatory bowel diseases

## Abstract

**Background:**

Microscopic colitis (MC) is suspected to result from increased immune activity in gut mucosa. Immune checkpoint inhibitors (ICIs) treat cancer by activating the immune system, and further investigation is needed regarding their role in the development of MC.

**Methods:**

A retrospective case series investigated cases of endoscopically and histologically confirmed MC developing after administration of ICIs. Clinical notes and medication administration records were reviewed for demographics, symptom duration, and treatment response.

**Results:**

Nineteen cases of de novo MC were identified, with 95% of cases requiring steroid treatment, 53% presenting with hospitalization, and colitis-related mortality in 1 individual. Symptom onset occurred a median of 160 days after initiation of ICI therapy and 53 days after their most recent dose of therapy. Patients had a median of 125 days of symptoms, and ICI therapy was held in 70% of individuals due for treatment.

**Conclusions:**

MC can develop after ICI administration, and presents with severe symptoms, often requiring hospitalization and steroid treatment. In certain individuals this can require a prolonged treatment course of steroid therapy or immunomodulators. Individuals developing diarrhea after ICI therapy warrant thorough workup including endoscopy and rapid treatment initiation given the disease severity observed in this series.

## Introduction

Immune checkpoint inhibitors (ICIs) have revolutionized the treatment of multiple cancer types; however, their use has been associated with immune-mediated complications, including gastrointestinal inflammation.^1^ Even though ICI-induced enterocolitis resembling inflammatory bowel disease is recognized, little is known regarding the development of ICI-induced colitis resembling microscopic colitis (MC).^2^ Although the etiology of MC is not fully understood, increased CD4- and CD8-positive T cells have been demonstrated in the bowel mucosa of MC patients.^3^

ICIs activate the immune system by promoting cytotoxic T-cell survival and enhancing tumor surveillance. They target programmed cell death protein 1 (PD-1) (nivolumab, pembrolizumab, and cemiplimab), PD-ligand 1 (atezolizumab, durvalumab, and avelumab), or cytotoxic T lymphocyte-associated protein 4 (ipilimumab). These medications can lead to autoimmune side effects, including ICI-induced colitis.^4^ Patients developing ICI-induced colitis can present without endoscopic findings, and in some of these cases, mucosal biopsies show findings consistent with a diagnosis of MC. Further research remains to delineate the characteristics of ICI-induced colitis resembling MC.

## Methods

Electronic medical records from January 2015 to October 2020 were reviewed across a tertiary medical center operating in 3 separate states to identify patients with International Classification of Diseases, 9th/10th revision, codes for MC, collagenous colitis, or lymphocytic colitis who had been prescribed ICI therapy (pembrolizumab, nivolumab, ipilimumab, atezolizumab, durvalumab, cemiplimab, and avelumab). Clinical and histology information was compared before and after ICI administration in patients who subsequently developed MC. Review of clinical notes was performed to assess symptom duration, treatment modalities, and clinical course of MC. Cases were excluded if MC had been diagnosed or symptoms preceded ICI receipt. Symptom remission was defined as improvement in diarrhea to <3 stools per day without relapse of symptoms over steroid taper. Refractory disease was defined as therapeutic steroid dose required for >6 months without ability to tolerate taper. Patients who started medications with a high likelihood to cause MC (statins, nonsteroidal anti-inflammatory medications, proton pump inhibitors, selective serotonin inhibitors) within 2 months of symptom onset were excluded. Individuals with symptom onset greater than 1 year after ICI receipt were also excluded.

MC diagnosis was confirmed by endoscopic evaluation and biopsy to rule out other forms of colitis such as ulcerative or Crohn’s colitis. Lymphocytic or collagenous colitis diagnosis was determined by pathological interpretation of random colonic biopsies. The diagnosis of lymphocytic colitis was based on the standard of 20 intraepithelial lymphocytes (IEL) per 100 surface epithelial cells on histologic evaluation. The diagnosis of collagenous colitis was determined by increased lymphocyte predominance compared to normal (but less than the 20 IEL/normal epithelial cells ratio seen in lymphocytic colitis) with the addition of a thickened collagen band (>7 µM, compared to the upper limit of normal being 5 µM). Additionally, while features of active inflammation such as neutrophil presence and active cryptitis could be present, they were not the predominant inflammatory infiltrate.

### Ethical Considerations

This is a retrospective analysis of anonymized data. We attest that this draft is free from plagiarism and the authors are in accordance with Crohn’s and Colitis 360’s ethical standards.

## Results

A total of 19 cases of patients diagnosed with de novo MC post-ICI therapy were identified. Sixteen patients (84%) had lymphocytic colitis and 3 (16%) had collagenous colitis. Eleven patients (58%) were diagnosed by colonoscopy and 8 (42%) by flexible sigmoidoscopy. Eight patients (42%) underwent colorectal cancer screening colonoscopies prior to symptom onset, and none of the colonoscopies had evidence of macroscopic colitis. None of the prior colonoscopies contained random biopsies to assess for MC. Two patients were current smokers (11%).

Pembrolizumab (47%) and nivolumab (26%) monotherapies were the most frequent ICI used, and 21% of cases received multiple ICIs. The most common ICI combination was nivolumab and pembrolizumab (*n* = 2, 11%). A total of 53% of cases were hospitalized at the time of MC diagnosis. ICI therapy was held due to colitis in 70% of the cohort who had yet to complete ICI treatment course for their malignancy. The most common malignancy prompting ICI treatment was melanoma (*n* = 10), followed by nonsmall cell lung carcinoma (*n* = 4).

The median time from first ICI treatment to symptom onset was 160 days (interquartile range (IQR), 95–281), with the most recent ICI dose given a median of 53 days prior (IQR, 45–66). Providers tended to initiate symptomatic treatment prior to endoscopic diagnosis. The median time to treatment initiation after symptom onset was only 22 days (IQR, 8–45); however, endoscopic diagnosis occurred a median of 84 days (IQR, 30–140) after symptom onset. Patients had a variable response to treatment, with a median of 125 days (IQR, 75–213) to symptom resolution after starting treatment.

The severity of colitis ranged from National Cancer Institute Common Terminology Criteria for Adverse Events V5.0 (CTCAE) diarrhea of grades 1–5 (Table 1).

**Table 1.** Characteristics of individuals developing de novo microscopic colitis after immune checkpoint inhibitor treatment (*n* = 19).

A. Demographics	
Median age, years (IQR)	66 (56–75)
Female	8 (42%)
Never smoker	8 (42%)
Former smoker	9 (47%)
Current smoker	2 (11%)
B. Histologic diagnosis	
Biopsies via colonoscopy	11 (58%)
Biopsies via flexible sigmoidoscopy	8 (42%)
Lymphocytic colitis	16 (84%)
Collagenous colitis	3 (16%)
C. ICI subtypes	
Pembrolizumab	9 (47%)
Nivolumab	5 (26%)
Atezolizumab	1 (5%)
Nivolumab + pembrolizumab	2 (11%)
Nivolumab + ipilimumab	1 (5%)
Ipilimumab + pembrolizumab	1 (5%)
D. Malignancy	
Melanoma	10 (53%)
Nonsmall cell lung	4 (21%)
Genitourinary	2 (11%)
Head and neck	2 (11%)
Small cell lung	1 (5%)
E. Impact of disease	
ICI treatment held	7 (70%)^a^
Hospitalized for diagnosis	10 (53%)
Refractory disease requiring long-term treatment	3 (16%)
Colitis-related mortality	1 (5%)
F. MC treatment medications	
Systemic steroids	13 (68%)
Enteric steroids	12 (63%)
Both systemic and enteric steroids	7 (37%)
Antidiarrheal alone	1 (5%)
Biologics	1 (5%)
Achieved steroid-free remission^b^	13 (68%)
Average number of treatments, *n* (SD)	2.2 (0.81)
G. Timing of MC development	
Median days from first ICI dose to symptom onset, days (IQR)	160 (95–281)
ICI treatment continued after symptom onset	9 (47%)
Median time from last ICI dose to symptom onset, days (IQR)^c^	53 (45–66)
Median time from symptom onset to endoscopic diagnosis, days (IQR)	84 (30–140)
Median time from symptom onset to treatment initiation, days (IQR)	22 (8–45)
Median symptom duration after treatment initiation, days (IQR)^d^	125 (75–213)
Total duration of symptoms, days (IQR)^d^	210 (94–256)
H. CTCAE grading of diarrhea	
1	2 (11%)
2	6 (33%)
3	8 (42%)
4	2 (11%)
5	1 (5%)
I. Additional ICI toxicity	5 (26%)

Values listed as *n* (%) unless otherwise specified. Abbreviations: ICI, immune checkpoint inhibitor; IQR, interquartile range; MC, microscopic colitis; *N*, number of patients. CTCAE, National Cancer Institute Common Terminology Criteria for Adverse Events. Version 5.0 diarrhea. Grade 1 = increase of <4 stools per day over baseline. Grade 2 = increase of 4–6 stools per day over baseline or limiting instrumental activities of daily living. Grade 3 = increase of 7+ stools per day over baseline, or hospitalization, or limiting self-care activities of daily living. Grade 4 = life threatening consequences requiring urgent intervention or ICU level cares. Grade 5 = death.

Treatment held in 7/10 individuals who were still due for ICI therapy.

Individuals who expired within 2 months of symptom onset excluded.

Individuals who received ICI despite symptom development excluded.

Individuals requiring persistent steroid treatment for >6 months or who never received symptom improvement were excluded from calculations regarding symptom duration.

Eighteen individuals (95%) received some form of steroid treatment. Thirteen (68%) required systemic corticosteroid therapy, 12 (63%) received enteric steroids, and 7 (37%) received both systemic and enteric steroids over the course of their disease. Only 1 individual received vedolizumab, which induced remission. Steroid-free remission was achieved in 13 patients (68%), while 3 (16%) were still steroid-dependent 6 months following onset of MC. One patient died due to electrolyte abnormalities from colitis. Another patient died 2 months after diagnosis with failure to thrive secondary to ICI-induced enteritis.

Additional ICI toxicities were identified in 5 individuals in this cohort. They included pneumonitis, pancreatitis, hypophysitis, thyroiditis, and peripheral motor neuropathy. All the toxicities were CTCAE grade 2 except for the peripheral motor neuropathy which was CTCAE grade 3.

## Discussion

We describe the largest series of patients diagnosed clinically with ICI-induced colitis with the histologic appearance of MC and make a novel observation that these patients have severe symptoms often requiring hospitalization. Of the 19 individuals who developed MC, 95% required steroids, 58% had severe colitis with CTCAE grading of diarrhea of 3 or greater, 53% required hospitalization, 1 individual died from enteritis, and 1 individual died from complications related to colitis. The overall colitis-associated mortality rate in our cohort was 5%. In addition, 26% of this cohort suffered additional ICI toxicities. The small size of this subgroup limits our analysis for predisposing factors. Future studies are needed to better understand the risk factors for ICI toxicity.

**Figure 1.**
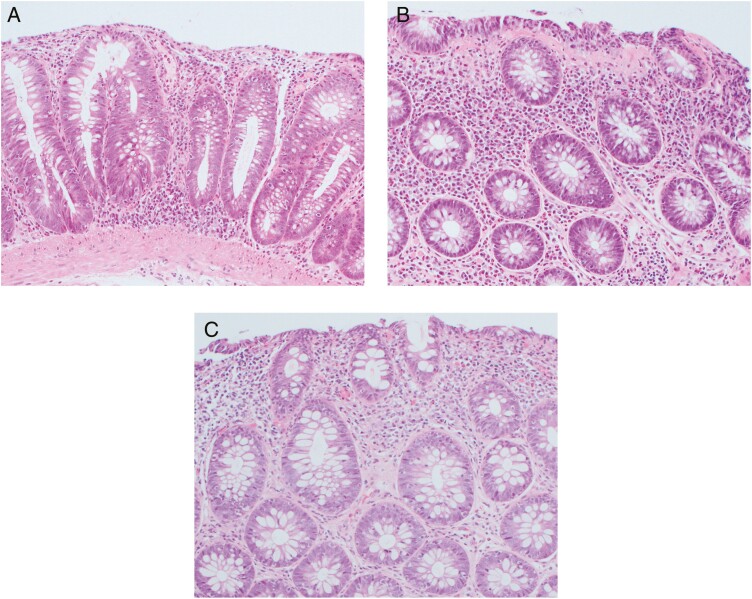
Histologic specimen stained with hematoxylin and eosin from individuals developing acute colitis after immune checkpoint inhibitor treatment. A, Findings displaying focal cryptitis, mildly increased intraepithelial lymphocytes, mildly focal increased crypt apoptosis, and focal Paneth cell metaplasia consistent with drug-induced acute colitis. B, Findings displaying increased intraepithelial lymphocytes with a thickened subepithelial collagen band representing collagenous colitis. C, Findings displaying increased intraepithelial lymphocytes (>20 cells per 100 surface epithelial cell) consistent with lymphocytic colitis.

While ICI-induced diarrhea and colitis have been extensively documented, de novo MC has not been well described.^5^ The diagnosis of MC differs from classically defined ICI-induced colitis, both endoscopically and histologically. Findings in acute colitis will show a diffuse inflammatory infiltrate with alteration of normal tissue as evidenced by neutrophilic abscesses, cellular apoptosis, and crypt destruction (Figure 1A). However, this differs from the thickened collagen band seen in collagenous colitis (Figure 1B) or the pure increase in IEL ratio (Figure 1C) seen with MC. In addition, the mucosa is typically endoscopically normal in appearance in patients with MC. In contrast, patients with ICI-induced colitis often have endoscopically confirmed inflammation characterized by erythema, friability, and ulceration. Our study shows that ICI-induced MC appears more severe than traditional MC in several respects. First, the disease presentation is more severe. Traditional MC has a much lower rate of hospitalization at the time of diagnosis, with a 7% rate seen in a recent cohort study of MC.^6^ ICI-induced MC also appears more refractory to treatment. This cohort’s median time to symptom remission was 125 days, even with 63% of individuals receiving budesonide, while previous studies describe improvement in 10–30 days in patients with MC treated with budesonide.^7^ This slower response may be due to the immune system activation after ICI administration making traditional treatments less effective. In addition, our cohort found a relatively equal distribution between males and females, whereas previous reviews have found a significant female dominance.^8^

There were some similarities in our cohort to classically defined MC. The cohort study by Loreau et al also found a diagnostic delay of 8 weeks, which is similar to our cohort.^6^ However, in our patients, treatment was often initiated prior to endoscopic diagnosis, likely due to closer observation from their oncologists.

While these patients were all diagnosed clinically and on histologic analysis with MC, their disease perhaps represents an intermediary between ICI-induced colitis more generally and MC. The histologic diagnosis of MC relies on the presence of >20 IEL per 100 epithelial cells seen on colonic biopsies. ICI-induced colitis typically has a patchier lymphocytic infiltrate that would not meet the criteria for MC.^9^ Given the lack of a respective control group we cannot draw any conclusions as to which risk factors predispose this population to developing MC in response to ICIs. Previous studies identified that patients experiencing MC trended toward exposure to a single agent PD-L1 compared to individuals developing classical acute colitis, although this did not reach statistical significance.^7^ Additional factors that may predispose individuals to developing a MC in response to ICI therapy include the relatively low percentage of current and former smokers in our population. Regardless, these patients all presented with a diagnosis of MC distinct from classically defined ICI colitis due to both their clinical and histologic appearance.

## Conclusion

In summary, we describe a case series of patients developing de novo MC after initiation of ICI therapy that appears more severe than classically described MC. These individuals often present with severe diarrhea requiring hospitalization. Individuals on ICI therapy presenting with symptoms of colitis warrant further diagnostic workup regardless of endoscopic findings including biopsies and prompt therapy given the disease course seen in this cohort. Further studies with larger sample size and prospective design are warranted to confirm our observations.

## Data Availability

The data that support the findings of this study are available from the corresponding author, L.E.R., upon reasonable request.
